# Delayed presentation of cerebellar and spinal cord infarction as a complication of computed tomography-guided transthoracic lung biopsy: a case report

**DOI:** 10.1186/1752-1947-8-272

**Published:** 2014-08-11

**Authors:** Kyung Min Shin, Jae-Kwang Lim, Chang-Ho Kim

**Affiliations:** 1 Department of Radiology, Kyungpook National University, School of Medicine, 680 Gukchaebosang-ro, Jung-gu, Daegu 700-842, South Korea; 2 Department of Internal Medicine, Kyungpook National University, School of Medicine, 680 Gukchaebosang-ro, Jung-gu, Daegu 700-842, South Korea

**Keywords:** Air embolism, Lung biopsy, Complication

## Abstract

**Introduction:**

Computed tomography-guided transthoracic needle biopsy is a common diagnostic procedure that is associated with various complications including pneumothorax, parenchymal hemorrhage, and hemoptysis. A systemic air embolism is a very rare (0.06 to 0.21%) but potentially fatal complication.

**Case presentation:**

A 70-year-old Korean male was admitted to our hospital for evaluation of a solitary pulmonary nodule located adjacent to the right inferior pulmonary vein in the medial basal segment of the right lower lobe. A computed tomography-guided needle biopsy was performed by a radiologist using a coaxial needle. A computed tomography image obtained immediately after the biopsy showed intraluminal free air in the proximal ascending aorta. He complained of a mild electrical current sensation in both lower extremities. After three hours he complained of neurological deficit in both lower extremities as well as voiding difficulty. The brain and spine magnetic resonance images showed a right cerebellar and spinal cord infarction at the T8-10 levels.

**Conclusions:**

We report a case of air embolism to the cerebellum and spinal cord causing infarction presenting with an initial symptom of mild electrical current sensation in both lower extremities during the transthoracic needle biopsy. For this potentially fatal complication, early recognition, followed by prompt therapy is critical to reducing morbidity and mortality.

## Introduction

Computed tomography (CT)-guided transthoracic needle biopsy is commonly performed to evaluate pulmonary and mediastinal lesions. While it is generally a safe method, there are reports of complications from this procedure. These complications include pneumothorax, intrapulmonary hemorrhage, hemoptysis, air embolism, seeding of the biopsy tract, and death. As a complication, air embolism is extremely rare, but can be fatal. Presently, we report a case of an air embolism in the cerebellum and spinal cord after a CT-guided transthoracic lung biopsy.

## Case presentation

**Figure 1:**
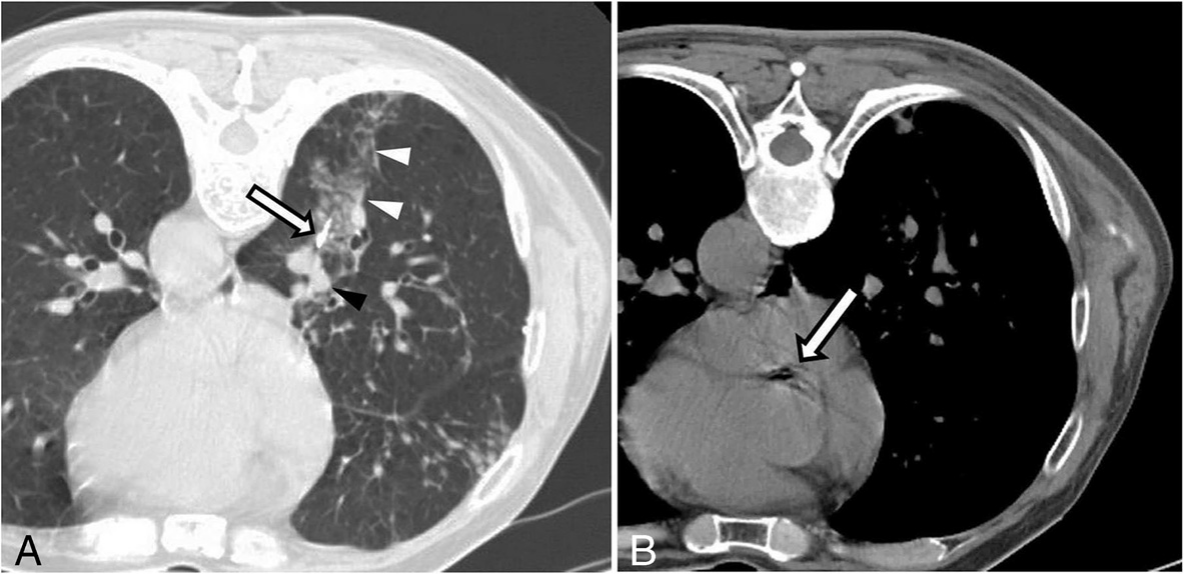
**Computer tomography images during and after transthoracic needle biopsy. (A)** Prone-positioned CT image obtained during the biopsy shows the biopsy needle (arrow) adjacent to the nodule and parenchymal hemorrhage along the route of the needle (white arrowheads). The pulmonary vein (black arrowhead) is located adjacent to the nodule. **(B)** CT image obtained immediately after the biopsy shows intraluminal free air in the proximal ascending aorta (arrow).

**Figure 2:**
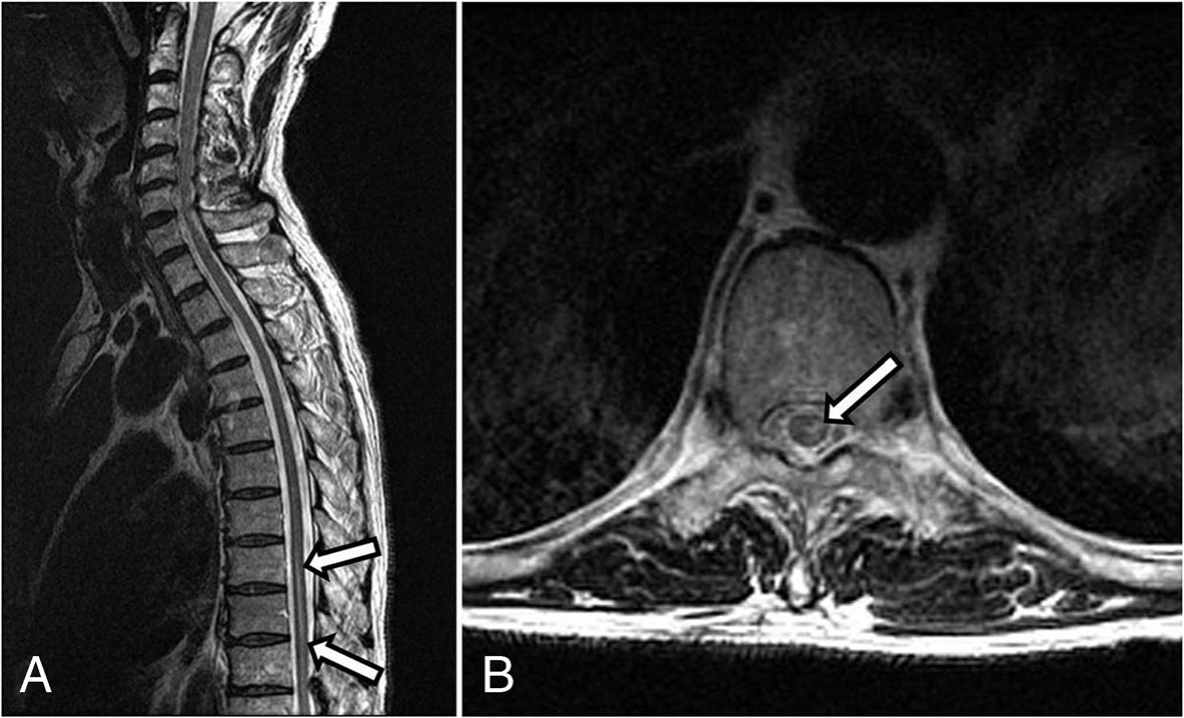
Axial (A) and coronal (B) T2-weighted magnetic resonance images show increased signal intensity (arrows) in the anterior spinal cord at the T8-10 levels.

**Figure 3:**
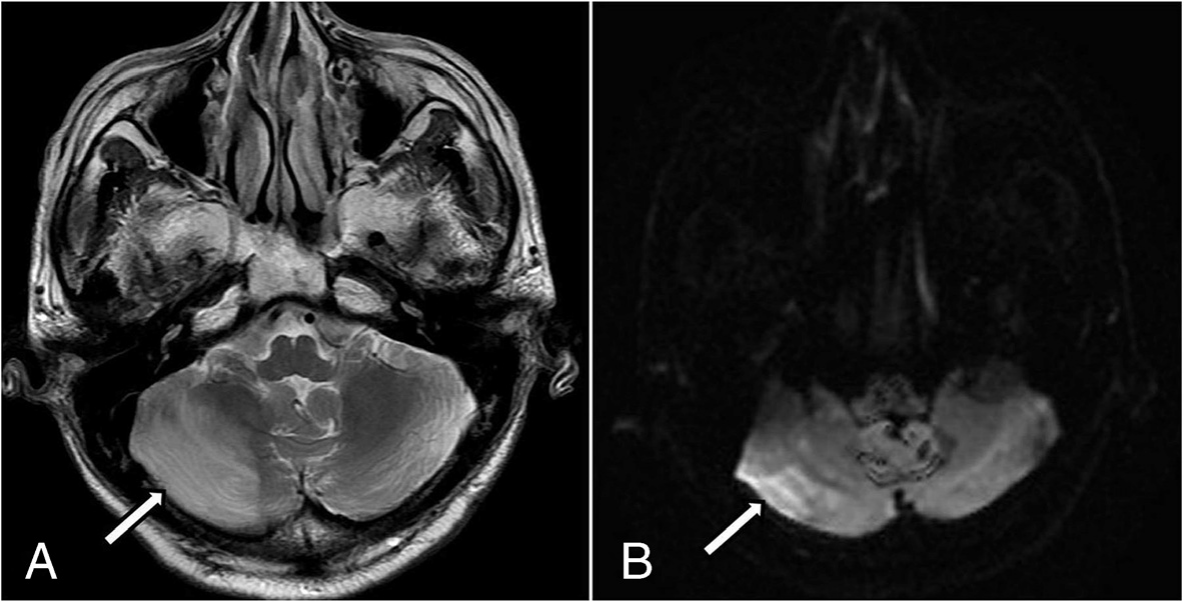
**(A) Axial T2-weighted magnetic resonance image shows diffuse high signal intensity in the right cerebellum (arrow). (B)** Diffusion-weighted magnetic resonance image at the same level demonstrates an increased diffusion restriction in the same region (arrow).

A 70-year-old Korean male patient was admitted to our institution with a medical history of mycobacterium infection and chronic obstructive pulmonary disease. He underwent repeated CT scans because of a solitary pulmonary nodule that was incidentally detected. A recent CT scan showed a 1.2cm sized, irregularly shaped nodule that was located adjacent to the right inferior pulmonary vein in the medial basal segment of the right lower lobe that had been increasing in size during the previous six months. A CT-guided needle biopsy was requested by a pulmonologist. After written informed consent was obtained, the biopsy was performed in the radiology department under CT guidance.Our patient was placed in a prone position and the lesion was localized with a CT scan. An 18-gauge coaxial needle (ACN Automatic Cutting Needle, Medical Device Technologies, Gainesville, Florida, United States) was inserted transthoracically during a single inspiratory breath-hold, and a core specimen was obtained using a biopsy gun (Figure 1A). Our patient was entirely cooperative during the procedure, refraining from coughing or deep breathing. After the removal of the needle, a post-procedural CT showed intraluminal free air in the ascending aorta (Figure 1B) as well as a small amount of parenchymal hemorrhaging along the needle path. He complained of a mild electrical current sensation in both lower extremities, but there was no neurological deficit revealed by physical examination, such as weakness or changes in sensory perception. The radiology operator did not recognize the intraluminal free air in the aorta because of the small amounts. Approximately three hours after returning to the ward, he complained of numbness and muscle weakness in both lower extremities, as well as voiding difficulty. The brain and spine magnetic resonance images (MRI) showed a right cerebellar and spinal cord infarction at the T8-10 levels (Figures 2 and 3). 100% oxygen was immediately administered via a facemask. He was stabilized and then transferred to a regional hospital for hyperbaric oxygen therapy. Following treatment, his neurological deficit and voiding difficulty were fully recovered after one month. A histopathological analysis revealed that he had small cell lung cancer.

## Discussion

CT-guided transthoracic lung biopsy is a minimally invasive and generally safe procedure that is used for the diagnosis of benign and malignant diseases that avoids the need for an exploratory thoracotomy. There have been many reports concerning various complications due to this procedure. The most frequent complication is pneumothorax, accounting for 27% of cases, followed by pulmonary hemorrhage (11%), and hemoptysis (7%) [1]. Systemic air embolism is a rare but potentially fatal complication, with a reported incidence of 0.06 to 0.21% [1-3].

Mansour *et al*. [4] listed three mechanisms whereby air can enter the systemic circulation during a needle lung biopsy. First, there is communication between the atmosphere and the pulmonary vein when the needle tip is placed within the pulmonary vein and the stylet has been removed. Second, when the needle passes through the lung parenchyma, a bronchovenous fistula may be created. There may be communication between intra-alveolar or intra-bronchial air and the pulmonary vein when the airway pressure is elevated, such as during a Valsalva maneuver, coughing, or positive pressure ventilation. Third, air in the pulmonary arterial circulation may reach pulmonary venous circulation by traversing the pulmonary microvasculature. Once in systemic circulation, an air embolus can lodge in any distal vessel, and the clinical presentation is similar to thromboembolic stroke syndrome or myocardial infarction, as has been previously reported [4-6]. The emboli tend to enter the vertebral artery through the brachiocephalic artery and descending thoracic aorta because the patient takes a prone position. The interesting feature of our case is that, to the best of our knowledge, this is the first report of a coexisting cerebellar and spinal cord infarction resulting from a transthoracic lung biopsy.

Regarding the available therapies for a systemic air embolism, the primary treatment involves supplying 100% oxygen, thereby promoting the replacement of nitrogen by oxygen and thus facilitating the resorption of the air. Positional therapy, such as the Trendelenburg position or right lateral decubitus, may be more helpful in cases of shock induced by a left ventricular air embolus before it causes an embolism in the brain [5,7]. Because the volume of gas in an enclosed space is inversely proportional to the pressure exerted on it, hyperbaric oxygen therapy reduces the size of air bubbles and accelerates the dissolution of nitrogen by replacing it with oxygen. In patients with cerebral air embolisms, the prompt initiation of hyperbaric oxygen therapy decreases morbidity and mortality [8].

A recent study by Hiraki *et al*. [9] reported that the incidence of systemic air embolisms may be underestimated by missing systemic air in patients without cardiac or cerebral symptoms. Radiologists must be aware of this extremely rare but potentially fatal complication so that they may be able to provide accurate diagnosis and treatment. It should be suspected in patients who present any neurological symptoms during the procedure. Several considerations, such as: avoiding needle biopsies of cystic, cavitary, or bullous lung parenchymas; penetrating the least amount of traversing lung parenchyma by choosing an appropriate entrance site to reach the mass; and performing the procedure by means of CT guidance are recommended to decrease the risk of this complication [7].

## Conclusions

We report the case of a development of an infarction in the cerebellum and spinal cord as a complication of CT-guided transthoracic needle biopsy with an initial symptom of mild electrical current sensation in both lower extremities during the procedure. For this potentially fatal complication, early recognition followed by prompt therapy is critical to reducing mortality and neurological sequelae. The biopsy operators should be aware of any neurological symptoms that may suggest the presence of systemic air embolism during the procedure.

## Consent

Written informed consent was obtained from the patient for publication of this case report and accompanying images. A copy of the written consent is available for review by the Editor-in-Chief of this journal.

## Abbreviations

CT: Computed tomography; MRI: magnetic resonance imaging.

## Competing interests

The authors declare that they have no competing interests.

## Authors’ contribution

KMS prepared the manuscript. JKL and CHK made a contribution to manuscript reviewing. All authors have read and approved the manuscript.
